# Art and Disease: A Solution Offered

**Published:** 1894-02-24

**Authors:** 


### Editor's Letter-Box.

[Our correspondents are reminded that prolixity is a great bar to publication, and that brevity of style and conciseness of statement greatly facilitate early insertion.]

### ART AND DISEASE—A SOLUTION OFFERED.

Dr. J. C. Webster writes from the University, Edinburgh: In your very interesting article on "Art and Disease" in The Hospital, February 10th, you refer to the error made by one of Raphael's pupils, who was depicted, in a fresco at the Vatican, "a helpless invalid with the muscles of a Hercules, and with limbs as stout as his body, and who, perfectly capable of supporting it, is dragging himself painfully along on his knees." Is it not possible that this might be a representation of a case of pseudo-hypertrophic paralysis, in which with great size limbs there is weakness? Such a case may have been seen by the painter.

In another short article in the same number you refer to the recent attention which has been paid to blindness caused by the action of low organisms. It may interest your readers to hear that one of our biological workers, Mr. G. S. Sandeman, has recently discovered wide-spread blindness among eels in a loch on the Island of May, at the mouth of the Forth. Microscopic examination has proved that the affection is due to the destruction of the cornea by the growth of coccidia in it.

